# Protective effects of ginseng extracts and common anti-aggregant drugs on ischaemia–reperfusion injury

**DOI:** 10.5830/CVJA-2015-047

**Published:** 2015

**Authors:** Ahmet Caliskan, Oguz Karahan, Yazici Suleyman, Demirtas Sinan, Guclu Orkut, Tezcan Orhan, Yavuz Celal

**Affiliations:** Department of Cardiovascular Surgery, Medical School of Dicle University, Diyarbakir, Turkey; Department of Cardiovascular Surgery, Medical School of Dicle University, Diyarbakir, Turkey; Department of Cardiovascular Surgery, Medical School of Dicle University, Diyarbakir, Turkey; Department of Cardiovascular Surgery, Medical School of Dicle University, Diyarbakir, Turkey; Department of Cardiovascular Surgery, Medical School of Dicle University, Diyarbakir, Turkey; Department of Cardiovascular Surgery, Medical School of Dicle University, Diyarbakir, Turkey; Department of Cardiovascular Surgery, Medical School of Dicle University, Diyarbakir, Turkey

**Keywords:** ginseng, herbal medicine, anti-aggregant drugs, ischaemia–reperfusion injury

## Abstract

**Objective:**

Ginseng is a traditional herbal medicinal product widely used for various types of diseases because of its cellular protective effects. Possible protective effects of ginseng were investigated in blood, cardiac and renal tissue samples and compared with common anti-aggregant agents in an animal ischaemia–reperfusion (I/R) model.

**Methods:**

Twenty rats were equally divided into four different groups as follows: control group (I/R-induced group without drug use), group I (acetylsalicylic acid-administered group), group II (clopidogrel bisulfate-administered group), group III (ginsenoside Rb_1_-administered group). For the groups assigned to a medication, peripheral I/R was induced by clamping the femoral artery one week after initiation of the specified medication. After reperfusion was initiated, cardiac and renal tissues and blood samples were obtained from each rat with subsequent analysis of nitrogen oxide (NOx), malondialdehyde (MDA), paraoxonase 1 (PON1) and prolidase.

**Results:**

NOx levels were similar in each group. Significant decrements were observed in serum PON1 levels in each group when compared with the control (*p* < 0.05). Serum MDA levels were significantly lower in groups II and III (*p* < 0.05). Ameliorated renal prolidase levels were detected in study groups (*p* < 0.05) and recovered cardiac prolidase levels were obtained in groups II and III (*p* < 0.05).

**Conclusion:**

These findings indicate that ginseng extracts may have a potential beneficial effect in I/R injury. However, more comprehensive studies are required to clarify the hypothetical cardiac, renal and systemic protective effects in reperfusioninduced oxidative damage.

## Objective

The meaning of the Chinese word ‘ginseng’ is ‘human seed’.Ginseng has a root-like appearance, and its extracts contain sponin. Ginseng has been used in traditional medicine for many years, especially in East Asian countries.^1^ So far, more than 30 ginsenosides have been defined.^2^,^3^ Ginsenoid-Rd [dammar-24(25)-ene-3β, 12β, 20(S)-triol-(20-O-β-D-glucopyranosyl)-3-O-β-D-glucopyranosyl-(1→2)-β-D-glucopyranoside] is one of the basic active substances of ginsenoids. Due to its antioxidant properties, it has been used in ischaemia–reperfusion experiments.^4^

Yokozawa *et al*. reported that ginseng had protective effects on rat models in ischaemia–reperfusion experiments.^5^ The protective effects of anti-aggregant drugs have also been reported in many ischaemia–reperfusion experiments performed on rats.^6^-^8^

The preparation of medicines and products containing ginseng varies from region to region and culture to culture. In traditional Chinese medicine, ginseng plants are harvested in their natural state, usually without being subjected to any further processing. In addition, they are only prepared by pulverisation so that they can be eaten with foods that are consumed daily. In modern medicine, ginsenoids obtained from ginseng plants are decomposed in such a manner that they can be used either in vitamin extracts or in hard gelatin capsules that contain a specified dose.^1^-^3^

This study was undertaken to evaluate the effects of ginseng extracts on ischaemia–reperfusion injury. Additionally, the protective effects of these extracts were compared with standard anti-aggregant drugs.

## Methods

Approval for this study was obtained from the local ethics committee and from the Animal Research Committee of Dicle University (2013/6). All procedures were performed according to the Animal Welfare Act and the *Guide for the Care and Use of Laboratory Animals*. All animal subjects were maintained at the laboratory of the Animal Production Unit at Dicle University in standard humidity- (50 ± 5%) and temperature- (22 ± 2°C) controlled cages with a 12-hour light/dark cycle until the study began.

Twenty rats were divided equally into four groups, including one control group. The rats in the control group underwent femoral ischaemia–reperfusion (I/R) without medication (the vehicle control-treated saline). These rats were sacrificed, and blood samples and cardiac and renal tissues were taken to determine the baseline I/R values of oxidative markers. All surgical procedures (without additional intervention) in the control group were designed similarly to the study groups. The ethics committee decided that there was no additional requirement for a sham group for determining the effect of surgical incision.

Three study groups were created in order to compare the protective roles of different agents. All rats were anesthetised with ketamine (Ketalar, Pfizer) at a dose of 130 mg/kg and xylasine (Rompun, Bayer) at a dose of 20 mg/kg via an intraperitoneal line. Maintenance of anaesthesia was provided with ketamine hydrochloride (50 mg/kg).

Three different agents were used for the three separate study groups before I/R, as follows. Group I (*n* = 5): acetylsalicylic acid (Coraspin®, Bayer, Leverkusen, Germany) was administered orally via gavage at a dose of 30 mg/kg/day, beginning one week prior to the start of the study. I/R was induced after one week of medication administration. Group II (*n* = 5): clopidogrel bisulfate (Planor®, Koçak Farma, Tekirdağ, Turkey) was administered orally via gavage at a dose of 1 mg/kg/day, beginning one week prior to the start of the study. I/R was induced after one week of drug administration. Group III (*n* = 5): ginsenoside Rb1 (Panax®, Bayer, Leverkusen, Germany) was administered orally via gavage at a dose of 100 mg/kg/day, beginning one week prior to the start of the study. I/R was induced after one week of drug administration.

## Experimental I/R injury modelling

**Fig. 1.**
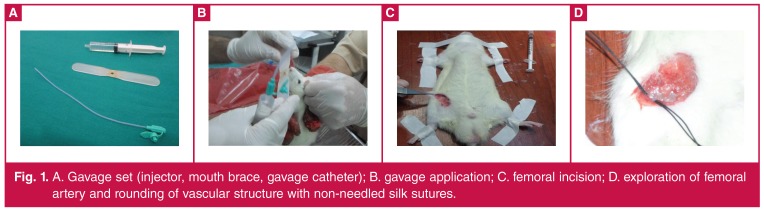
A. Gavage set (injector, mouth brace, gavage catheter); B. gavage application; C. femoral incision; D. exploration of femoral artery and rounding of vascular structure with non-needled silk sutures.

The right femoral arteries of all of the rats were explored with simple femoral incision and the femoral artery was rounded with a non-needle suture USP-3/0 metric silk (Dogsan Surgical Sutures, Medical Material Industry Co. Inc, Trabzon, Turkey); thereafter the femoral the artery was clamped for six hours (Fig. 1). The femoral clamp was removed to create reperfusion after six hours. After reperfusion, all rats were sacrificed in the first hour, and blood samples and cardiac and renal tissues were obtained from each rat in each group.

All study protocols were designed according to previously published protocols.^9^ The drug utilisation and surgical protocols are outlined in Fig. 1.

## Laboratory analyses

NOx measurement: the Griess reagent method, which is based on a modified cadmium reaction, was used to determine nitrogen oxide (NOx) levels. This method measures platelet-derived nitric oxide as described by Yavuz *et al*.^10^ NOx levels were calculated as μM/g protein for tissue extracts and as μmol/l for blood samples.

MDA measurement: malondialdehyde (MDA) levels were evaluated according to the method described by Ohkawa *et al*., which is based on the determination of the levels of thiobarbituric acid reactive products.^11^ MDA values were expressed as μM/g protein for tissue extracts and as μmol/l for blood samples.

PON1 measurement: the spectrophotometric modified Eckerson method was used for the detection of paraoxonase 1 (PON1) activity.^12^ The activity of PON1 was expressed as U/g protein for tissue extracts and as U/l for blood samples.

Prolidase measurement: prolin (expressed as U/l protein for tissue extracts and as U/g for blood samples), which is produced by prolidase, was measured spectrophotometrically according to the method described by Myara *et al*.^13^

## Statistical analysis

Oxidative markers in each group were analysed with SPSS software version 15.0 (SPSS Inc., Chicago, IL), and *p* < 0.05 was considered to be statistically significant. Obtained values were presented as mean ± standard deviation (SD). The Kolmogorov–Smirnov test was used to assess the normality of the distributions. Differences in the mean values between groups were assessed with a one-way analysis of variance (ANOVA) test and a Tukey HSD was used as a *post hoc* test; *p* < 0.05 was considered to be statistically significant.

## Results

In the control group, NOx levels were 8.99 ± 5.01 μmol/l, 25.36 ± 3.69 μM/g protein, and 14.06 ± 4.12 μM/g protein for blood, cardiac and renal samples, respectively. The control group’s MDA values were 24.63 ± 3.23 μmol/l, 28.38 ± 4.87 μM/g protein, and 13.11 ± 3.90 μM/g protein for blood, cardiac and renal samples, respectively. The activities of PON1 in the control group’s blood, cardiac and renal samples were 256.55 ± 19.06 U/l, 18.89 ± 7.41 U/g protein, and 20.75 ± 5.01 U/g protein, respectively. The control group’s prolidase levels were 1283.52 ± 545.44 U/l for blood samples, 63.47 ± 11.51 U/g protein for cardiac tissue extract, and 96.26 ± 4.12 U/g protein for renal tissue extract.

**Fig. 2.**
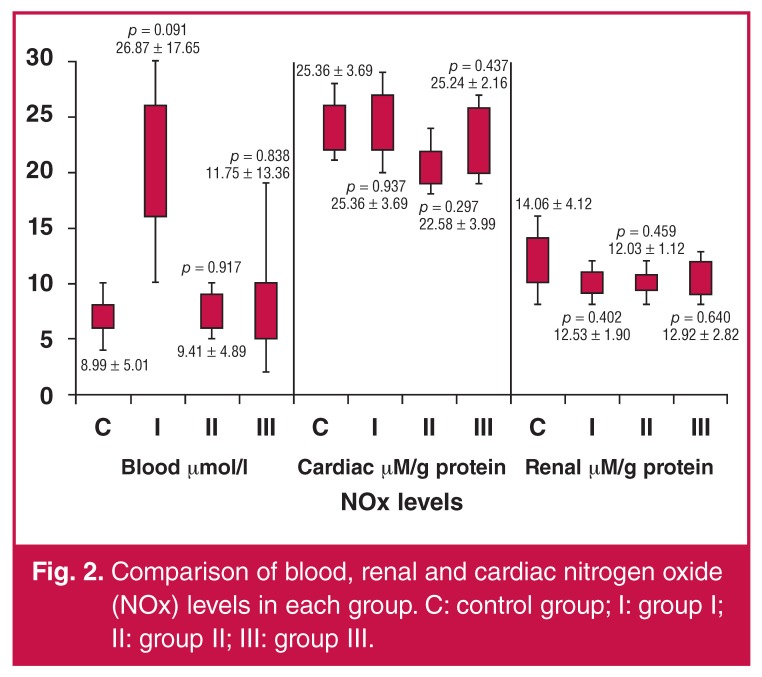
Comparison of blood, renal and cardiac nitrogen oxide (NOx) levels in each group. C: control group; I: group I; II: group II; III: group III.

There were no significant differences between the groups in terms of NOx levels. The comparison of NOx values between the groups is presented in Fig. 2.

The PON1 activity in the blood from each drug group was significantly different from that of the control group [control vs group I (acetylsalicylic acid), (*p* < 0.05); control vs group II (clopidogrel), (*p* < 0.05); control vs group III (ginsenoside), (*p* < 0.05)]. There was a significant difference (*p* < 0.05) between the control group and group III (ginsenoside) in terms of blood MDA levels. However, blood MDA levels of group II (clopidogrel) were significantly lower than those of the controls (*p* = 0.045). There were no significant differences between the control group and any of the study groups in terms of cardiac oxidative markers (*p* > 0.05).

**Fig. 3.**
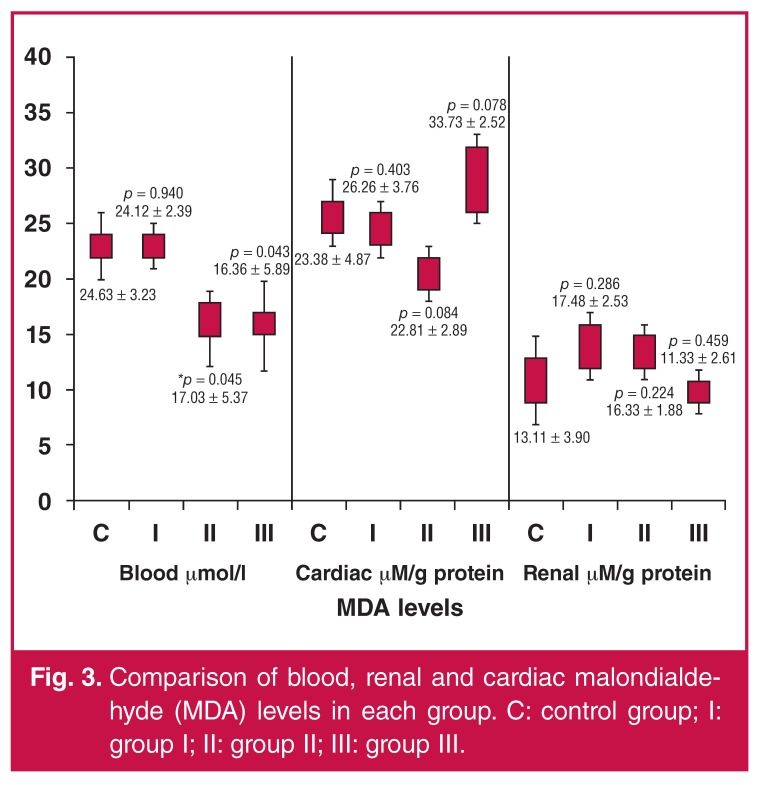
Comparison of blood, renal and cardiac malondialdehyde (MDA) levels in each group. C: control group; I: group I; II: group II; III: group III..

**Fig. 4.**
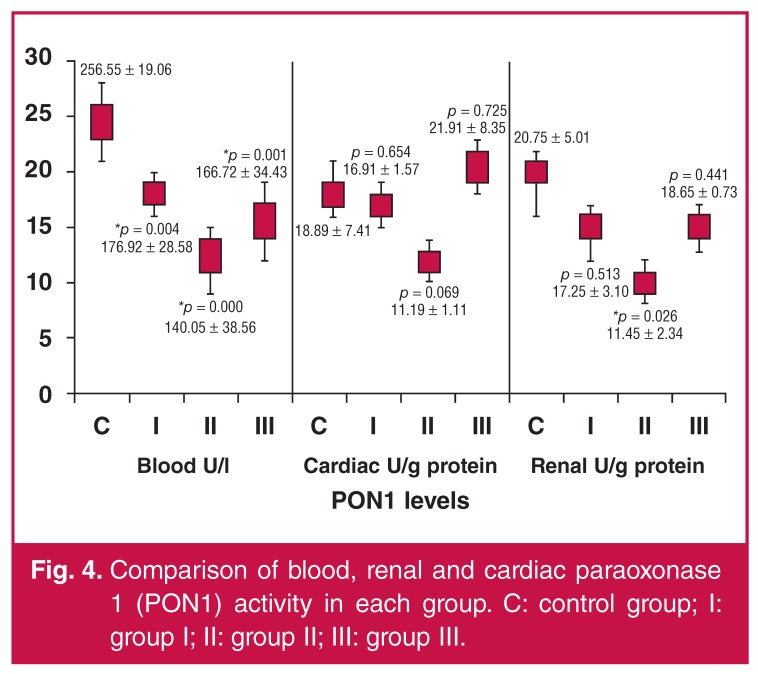
Comparison of blood, renal and cardiac paraoxonase 1 (PON1) activity in each group. C: control group; I: group I; II: group II; III: group III.

The MDA levels in each group are compared in Fig. 3. Both renal prolidase and PON1 levels were significantly lower in group II (clopidogrel) than in the control group (*p* < 0.05). Similarly, renal prolidase levels were also markedly lower in group III (ginsenoside, *p* < 0.05). The PON1 activities of each group are presented in Fig. 4.

**Fig. 5.**
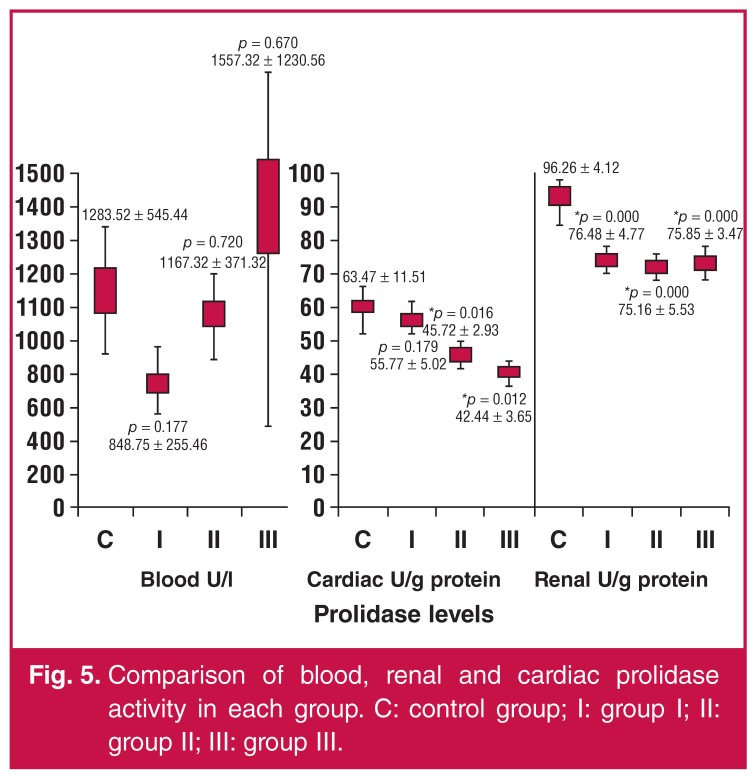
Comparison of blood, renal and cardiac prolidase activity in each group. C: control group; I: group I; II: group II; III: group III.

Renal prolidase levels were significantly lower in group I (acetylsalicylic acid) than in the control group (*p* < 0.05). In addition, cardiac prolidase levels were significantly lower in groups II (clopidogrel) and III (ginsenoside) (*p* < 0.05). Prolidase levels are compared between groups in Fig. 5.

## Discussion

Our results suggest that experimental I/R induced oxidative markers in blood, cardiac and renal samples. NOx values were similar in both the study and control groups. Blood MDA values were markedly lower in the clopidogrel and ginsenoside groups when compared with the control group. Decreased PON-1 levels were found in the clopidogrel group when compared with other groups. Significantly decreased cardiac prolidase levels were detected in the clopidogrel and ginsenoside groups when compared with the control group. Renal prolidase levels were markedly decreased in all study groups that were treated with acetylsalicylic acid, clopidogrel or ginsenoside.

For centuries, herbal products have been widely used to treat or alleviate the symptoms of many diseases. Moreover, some of these herbs are currently used for traditional disease management.^14^,^15^ According to the World Health Organisation (WHO) report, it is estimated that more than 80% of the world’s population is dependent on herbal medicine.^14^ Most believe that it is safe to use these natural products, although side effects, toxicity and adverse drug interactions have been reported.

Most drugs originated from or are derived from herbs. However, dosages and usable forms of the drugs must be investigated with expensive clinical trials before they become commercialised.^14^,^16^ Although there are conflicting reports, herbal products, while they still have risks, may be safer for prophylaxis and the treatment of diseases. In addition, they are inexpensive and readily available.^17^ The quality and quantity of herbal products may vary depending on seasonal and regional growing conditions, and may therefore have different effects.^18^ Supervision and quality control should therefore be required during their production.

Ginseng is a commonly studied therapeutic herbal product.^18^ The protective role of ginseng extracts with several metabolic mechanisms has been reported in cardiovascular events.^19^ It has been hypothesised that ginseng extracts may protect the cardiovascular system by acting as an antioxidant, antihypertensive, antidiabetic and antinociceptive agent.^15^,^19^

The protective effects of acetylsalicylic acid and clopidogrel bisulfate on ischaemia–reperfusion injury have been previously described.^6^,^7^,^20^,^21^ In addition, similar findings have been reported for ginsenosides.^22^ However, to our knowledge, there has been no definitive comparison of these three agents in the literature.

In this study, the systemic, cardiac and renal protective effects of the well-known anti-aggregant agents acetylsalicylic acid and clopidogrel bisulfate were compared with ginsenoside Rb_1_ (Panax) against oxidant stress in a peripheral ischaemia–reperfusion model.

MDA serves as a biomarker for detection of peroxidative damage in reperfused organs; it is a product of enzymatic and oxygen radical-induced lipid peroxidation.^9^ Reduced MDA levels have previously been reported with acetylsalicylic acid- or clopidogrel-treated patients in ischaemia–reperfusion studies.^23^,^24^

Although, PON-1 is mainly produced by the liver, it has been identified in other tissues such as the kidney, heart and brain.^25^ An inverse relationship was reported between PON-1 activity and antiplatelet agents.^26^

Prolidase is a marker for collagen metabolism that is related to increased levels of nuclear hypoxia-inducible factor-1 alpha (HIF-1).^9^,^27^ Increased prolidase levels were reported in acute ischaemic events.^27^

In the current study, the serum MDA levels were partially improved in the clopidogrel bisulfate and ginsenoside Rb_1_ groups, while the serum PON1 levels were markedly decreased in all three groups. Renal PON1 levels were only significantly expressed in the clopidogrel bisulfate group. Renal prolidase levels were significantly decreased in all groups compared to the control I/R group. Cardiac prolidase levels were significantly decreased in the clopidogrel bisulfate and ginsenoside Rb_1_ groups. According to our results, it appears that ginsenoside Rb_1_ had a beneficial effect on the oxidative stress induced by I/R by antioxidant mechanisms.

Mannaa *et al*. reported that Panax had a neuroprotective effect in acrylamide-induced neurotoxicity.^15^ In another study, Basha *et al*. reported the renoprotective effects of ginsenosides against oxidative stress in streptozotocin-induced diabetic nephrotoxicity in mice.^22^ In addition, it has been reported that ginsenosides can play a protective role in decreasing lipid peroxidation and ameliorating oxidative damage.^28^

Kim reported that ginsenosides have possible protective mechanisms in cardiovascular events.^19^ He described these mechanisms as follows:

Ginsenosides inhibit Ca^2+^ entry, and therefore may ameliorate cardiac function.^19^ However, acetylsalicylic acid can stimulate the Ca2+ entry pathways.^29^
Ginseng normalises blood pressure and improves blood circulation.^19^ Previous reports noted that acetylsalicylic acid and clopidogrel can alter blood flow in tissues.^30^
Ginsenoids can protect against myocardial damage via nitric oxide-mediated cardiac protection, antioxidant and intracellular calcium homeostasis, and attenuation of calcineurin activation.^19^ Similarly, some literature has suggested that clopidogrel and acetylsalicylic acid improve endothelial nitric oxide.^31^,^32^
Ginseng saponin has a protective role on endothelial cells via a cellular signalling pathway.^19^ Similar cellular mechanisms were reported for clopidogrel and acetylsalicylic acid.^33^,^34^
Ginseng has a cardiovascular protective role in inhibiting oxidative damage due to the prevention of reactive oxygen species generation.19 Also, the anti-oxidant effects of clopidogrel and acetylsalicylic acid have been described in previous reports.^7^, ^34^

There are some limitations that need to be addressed in this study. An experimental I/R model was created for this study in healthy rats. Therefore, our results are pertinent only to the rat model and these results should be confirmed in human subjects. The other limitation is that only oxidative markers were studied and PCR and Western blot analysis were not applied. Because of this, our findings are lacking in cellular reflections.

## Conclusion

Herbal medicine is still important for the majority of the world’s population. Traditional ginseng extracts may have beneficial effects on ischaemia–reperfusion injury. However, we caution that herbs should not replace traditional drugs. Ginseng can be beneficial as a drug supplement when controlled by healthcare organisations. In addition, future cardiovascular studies are needed to clarify the drug interactions and the proper dose of ginseng extracts.
